# Wearable Belt With Built-In Textile Electrodes for Cardio—Respiratory Monitoring

**DOI:** 10.3390/s20164500

**Published:** 2020-08-12

**Authors:** Emanuele Piuzzi, Stefano Pisa, Erika Pittella, Luca Podestà, Silvia Sangiovanni

**Affiliations:** 1 Department of Information Engineering, Electronics and Telecommunications (DIET), Sapienza University of Rome, via Eudossiana 18, 00184 Rome, Italy; stefano.pisa@uniroma1.it; 2 Department of Legal and Economic Sciences, Pegaso University, via di S. Pantaleo 66, 00186 Rome, Italy; erika.pittella@unipegaso.it; 3 Department of Astronautics, Electrical and Energetics Engineering (DIAEE), Sapienza University of Rome, via Eudossiana 18, 00184 Rome, Italy; luca.podesta@uniroma1.it (L.P.); silvia.sangiovanni@uniroma1.it (S.S.)

**Keywords:** bio-impedance measurement, biomedical measurement, cardio–respiratory monitoring, ECG recording, patient monitoring, plethysmography, wearable sensors

## Abstract

Unobtrusive and continuous monitoring of vital signs is becoming more and more important both for patient monitoring in the home environment and for sports activity tracking. Even though many gadgets and clinical systems exist, the need for simple, low-cost and easily applicable solutions still remains, especially in view of a more widespread use within everyone’s reach. The paper presents a fully wearable and wireless sensorized belt, suitable to simultaneously acquire respiratory and cardiac signals employing a single acquisition channel. The adopted method relies on a 50-kHz current injected in the subject thorax through a couple of textile electrodes and on envelope detection of the trans-thoracic voltage acquired from a couple of different embedded electrodes. The resulting signal contains both the baseband electrocardiogram (ECG) signal and the trans-thoracic impedance signal, which encodes respiratory acts. The two signals can be easily separated through suitable filtering and the cardio–respiratory rates extracted. The proposed solution yields performances comparable to those of a spirometer and a two-lead ECG. The whole system, with a realization cost below 100 €, a wireless interface, and several hours (or even days) of autonomy, is a suitable candidate for everyday use, especially if complemented by motion artifact removal techniques, currently under implementation.

## 1. Introduction

Continuous monitoring and diagnosis through non-invasive measurements of vital signs represent two concrete technological responses to the challenges of care personalization and cost containment posed by modern medicine. Real-time, continuous and non-invasive measurement of physiological signals represents a key element for numerous applications, from patient monitoring in hospitals to home therapy [1], medical assistance and surveillance for the elderly [2], and athletic performance monitoring [3]. Nowadays, measuring vital signs continuously and non-invasively can be an invaluable tool for physicians that can make timely decisions and better choices when long-term patient data are available, preventing diseases and enhancing quality of life whilst reducing the costs of health care [4]. In particular, continuous cardio–respiratory monitoring can be useful for early detection of deteriorating patients, and improve patient safety by reducing the incidence of critical events [5].

The gold standard for measuring breath activity is the spirometry that measures the amount (volume) and/or speed (flow) of air that can be inhaled and exhaled, assessing breathing patterns that identify specific conditions. The limitations of the test are that it is highly dependent on patient cooperation and effort, and cannot be used in a continuous way. For heart activity monitoring, the electrocardiogram is the adopted gold standard. It makes use of electrodes placed on the skin, that register the electrical activity related to the cyclic cardiac muscle depolarization and repolarization. 

By using the electromagnetic radiation in the microwave region, it is possible to monitor cardio–respiratory activity without any contact with the subject under examination and, consequently, to carry out non-invasive monitoring. The systems proposed in the literature for the remote monitoring of breath activity are mainly based on Doppler [6,7], ultra wideband (UWB) [8,9] and FMCW radar techniques [10,11]. Their major advantage is that, without the need for any cable or electrode, it is possible to locate the patient inside the room, and then, to measure his/her respiratory rate and heartbeat. However, this seems to be a valid choice for patients in a hospital environment or at home, but is not easily implementable in a wearable system, even though antennas designed to be used in contact with the body might be employed [12].

Approaches based on wearable systems are gaining great interest for medical applications. In the literature, different categories of sensors have been investigated that can be embedded into textiles [13].

A competitive method for monitoring respiratory behavior for the chest and abdomen regions is based on Fiber Bragg Grating (FBG) sensors, which have been investigated for their advantages, such as high sensitivity, magnetic resonance compatibility, and the capability of performing distributed measurements [14,15]. Other breath monitoring solutions, still based on optical fiber, are also being considered [16,17]. Piezo resistive strain sensors [18], paper-based humidity sensors [19], textile capacitive respiration sensors [20], accelerometers [21,22], resistive strain gauges [23,24], dew-based sensors [25,26], flow meters [21,27], and magnetic induction coils [28] are also largely considered in the literature. However, the above cited systems are able to monitor exclusively breath activity.

Recently, numerous wearable sensors for long-term human heart activity monitoring have been proposed [29,30,31]; however, only a few proposals for monitoring systems that allow a simultaneous detection of breath activity and heartbeat exist in the literature. In [32], an FBG system is used for a simultaneous monitoring of respiratory and cardiac activities. The results are very promising in terms of accuracy with respect to [33,34]. However, simultaneous breath and cardiac sensing can only be achieved in standing and supine positions. Fiber optic-based systems have also been used for heart rate monitoring in [35], but in this case the system has been assessed only in supine position and it is not wearable. In [36] a wearable FBG-based system for heart rate monitoring is presented but its measurement uncertainty has not been analyzed in detail. In [4] a simultaneous piezoelectric noninvasive detection that incorporates the cardiac and respiratory activities is developed, and the convolution theory with Fourier transform are applied to extract the corresponding cardiac activity signal from the respiratory signal. Measurements of electrocardiogram (ECG) and respiratory signals are presented [37] using capacitive sheet electrodes, but only when the individuals maintain ordinary body positions such as supine position and left and right lateral positions.

In [38] a T-shirt prototype that embeds novel textile sensors for the capture of cardio and respiratory signals is proposed. However, the need for two systems in a chest-band is declared, with two capacitive textile sensors to capture respiration signals, in addition to five non-removable skin-contact embroidery electrodes, for the ECG.

Together with the wide spectrum of sensing technologies above described for breath activity detection, a largely employed alternative is based on the measurement of bio-impedance, that allows gathering information on different vital parameters [39,40,41]. The principle of the impedance plethysmography is based on the recording of the trans-thoracic impedance, which changes proportionally to the volume of inspired air, through electrodes placed on the thoracic surface. If the trans-thoracic impedance measurement system has enough sensitivity, changes in the impedance related to the cardiac cycle can be detected [41,42,43,44]. This aspect suggests the possibility of designing a simple wearable cardio–respiratory monitoring device based on impedance plethysmography.

It is worth mentioning that an alternative solution for achieving simultaneous cardio–respiratory monitoring with a single sensor is to extract the breath signal from the ECG trace [45]. The feasibility of such approach has been demonstrated in the literature employing wavelets and empirical mode decomposition (EMD) [45], and a combination of EMD and principal component analysis (PCA) [46]. However, implementation of a wearable ECG device is slightly more complex, in terms of circuit realization, as compared to a bio-impedance plethysmograph.

In [41] the authors investigated the feasibility of a simple volt-amperometric bio-impedance measuring device for simultaneous detection of breath and cardiac activities. Starting from measurement of the basal trans-thoracic impedance on several volunteers it was possible to evaluate the sensitivity of the system, showing that the tetrapolar arrangement provides enough resolution for heartbeat detection. For ease of realization and testing the system was created through LabVIEW virtual instruments (VIs) based on a data acquisition (DAQ) card. The innovative aspect consisted in the fact that a portable cardio–respiratory monitoring, entirely based on impedance plethysmography, was implemented.

In this paper, a fully wearable version of the device is presented, where the pre-gelled self-adhesive electrodes employed in [41] are replaced with textile electrodes for greater comfort and ease of use. Secondly, low-power off-the shelf components for the entire device are used making the system low cost and self-contained in a thoracic belt. A wireless interface has been also added, allowing real-time monitoring and recording without any restraint to the patient mobility. Thanks to the low-power components the whole belt is fed by a couple of AAA alkaline batteries with a duration of several hours. Finally, a modification of the scheme originally proposed in [41] allows the device, with no addition in hardware complexity, to directly retrieve the ECG signal as a more robust and sensitive alternative to impedance plethysmography for detecting heartbeats. The developed system has the novelty, as compared to currently available solutions, that the respiratory and heart signals are acquired from a single channel where the ECG and impedance plethysmography signals are superimposed and later separated with proper filtering after envelope detection. Just as a comparison, there are even commercial solutions available (e.g. ADS1292R from Texas Instruments) which allow simultaneous ECG and respiratory signal acquisition. However, they employ two independent channels and two separate converters for acquiring the two signals.

## 2. Materials and Methods

**Figure 1:**
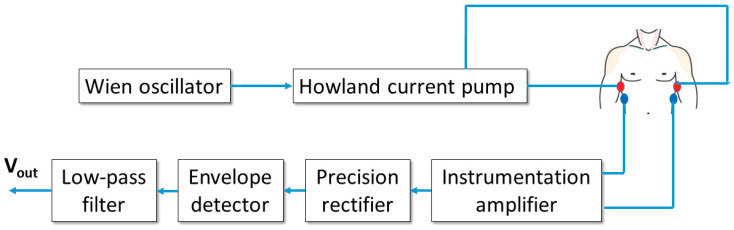
Scheme of the wearable instrument implementing a single acquisition channel able to simultaneously acquire impedance plethysmography and ECG signals.

As discussed in the Introduction, starting from the previous virtual instrument presented by the authors in [41], a fully-wearable and wireless instrument has been developed. The scheme of the device is reported in Figure 1.

### 2.1. Single-Channel Impedance Plethysmography and ECG Acquisition System

The basic scheme in Figure 1 allows for a 4-electrode bio-impedance measurement on the subject thorax, similarly to the virtual instrument in [41].

The Wien oscillator provides a sinusoidal signal with a frequency of about 50 kHz and a peak-to-peak amplitude of approximately 4 V. The oscillator feeds a voltage-to-current converter (Howland current pump), which outputs a sinusoidal current with a peak amplitude of about 0.5 mA. This level corresponds to an RMS amplitude of approximately 0.35 mA, well within the regulatory limits for patient current which, according to [47], must not exceed the 5 mA RMS level at the operating frequency of 50 kHz. The generated current is injected in the patient thorax through a first pair of electrodes (current electrodes).

A second pair of electrodes (voltage electrodes) picks up the voltage from the thorax surface. It must be stressed that such voltage includes two contributions: a 50-kHz signal, induced by the injected current, whose amplitude is modulated by the changes in the thoracic trans-impedance (which, as demonstrated in [41], are related to both respiratory and cardiac activities) and a baseband ECG signal directly generated by heart electrical activity. The voltage is detected by an instrumentation amplifier with a gain equal to 100. The amplified signal is then rectified through a precision rectifier (with unity gain and voltage inverting configuration) and processed with an envelope detector with a time constant equal to 10 ms. Such a time response is slow enough to filter the 50-kHz ripple and fast enough to accurately follow low-frequency variations due to breathing and heart activities. The detected signal is finally filtered through a second-order low-pass Sallen–Key filter with unity gain and a cutoff frequency of 12 Hz. The chosen cutoff frequency allows to filter out any residual 50-kHz ripple but also strongly attenuates possible 50-Hz interference resulting from unwanted coupling with the electrical supply network. Obviously a 12-Hz cutoff frequency will also eliminate high-frequency components from the ECG signal, but this not a real limitation since the purpose of the instrument is not to provide a clinical grade ECG signal, but a simpler two-lead ECG signal useful to reliably detect the cardiac frequency.

### 2.2. Simultaneous Impedance Plethysmography and ECG Acquisition

As detailed in the previous subsection, the voltage signal acquired by the instrumentation amplifier contains a 50-kHz amplitude modulated signal, which encodes information on the time-varying trans-thoracic impedance, and a baseband ECG component.

**Figure 2:**
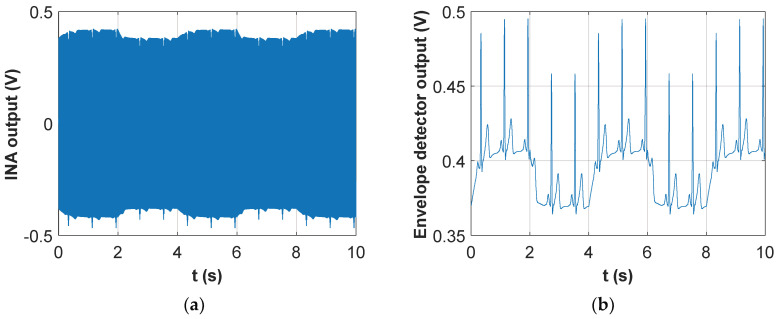
Synthetic example of a signal detected on the patient thorax through the scheme depicted in Figure 1: (**a**) Total voltage recorded across the thorax; (**b**) Output of the detection circuit after inverting rectification and envelope extraction.

An example of such a signal is represented in Figure 2a, where two synthetic signals have been added: an amplitude modulated carrier, where the modulating signal resembles the classic trans-thoracic impedance variation resulting from respiratory activity (in particular, three inspiratory and two expiratory acts are visible in the figure), and a baseband ECG signal, which has been considered “inverted” (i.e. the positive electrode is placed on the right side of the thorax and the negative one on the left side). The reason for considering an “inverted” ECG signal is to retrieve it with the right polarity after being processed by the precision rectifier, which exploits an inverting configuration. 

Figure 2a clearly shows that the baseband ECG signal does not perform an amplitude modulation of the carrier (it does not produce a symmetrical variation of the signal envelope, as opposed to the trans-thoracic impedance). On the contrary, the ECG signal acts as a time-varying offset added to the breath-modulated signal. Nonetheless, the final result is that the ECG signal is clearly visible on the negative envelope of the total signal, which will be detected by the proposed circuit. In fact, Figure 2b shows the result of processing the signal in Figure 2a with the combination of the inverting precision rectifier and envelope detector. The detected signal clearly contains both the effect of respiratory and cardiac activities. As a side note, it is important to stress that the actual signal retrieved from the patient thorax will also show the effect of cardiac activity on the trans-thoracic impedance signal, as already demonstrated in [41]. However, it will be shown in Section 2.5 that the possibility of directly detecting the baseband ECG signal results in a much more accurate and reliable estimation of the heart rate of the subject. It is also evident from Figure 2b that the two physiological signals (i.e. respiration and heartbeat) are characterized by substantially distinct time scales, so that their separation through simple filtering, rather than more complex signal processing solutions such as empirical mode decomposition (EMD) [45,46], is straightforward. The effectiveness of frequency selective filtering for separating the two signals will be demonstrated in Section 3.

### 2.3. Realization of the Sensorized Belt with Textile Electrodes

In order to realize a fully wearable device, the scheme depicted in Figure 1 has been implemented on a PCB with very compact size using surface-mount device (SMD) components. The board layout has been designed using Easily Applicable Graphical Layout Editor (EAGLE) electronic design automation software (Autodesk) and has been realized as a double layer process through standard chemical etching.

**Figure 3:**
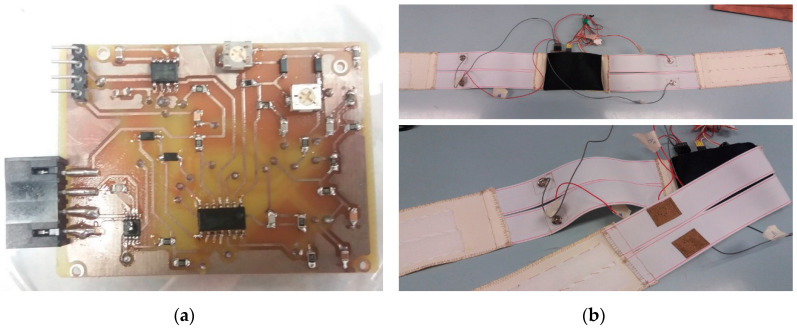
Prototype of the sensorized belt: (**a**) PCB for respiratory and cardiac signal acquisition; (**b**) Stretchable belt with textile electrodes and pocket for battery pack, PCB and micro-controller placement.

Figure 3a shows a picture of the top (component) side of the developed board, with the components soldered in place.

The whole board is powered by two AAA batteries providing a total voltage of about 3 V. In order to obtain a balanced supply, a TC7662B charge-pump DC–DC voltage converter (Microchip Technology, EW CBS, Nashik 422002, Maharashtra, India) is mounted on the board, providing the—3 V rail. The Wien oscillator, Howland current pump, precision rectifier and Sallen–Key filter use each one operational amplifier from a LM6134 quad low-power rail-to-rail chip (Texas Instruments). Finally, an AD8220 rail-to-rail instrumentation amplifier (Analog Devices, One Technology Way, P.O. Box 9106, Norwood, MA 02062-9106, USA) is employed for detecting the trans-thoracic voltage.

**Figure 4:**
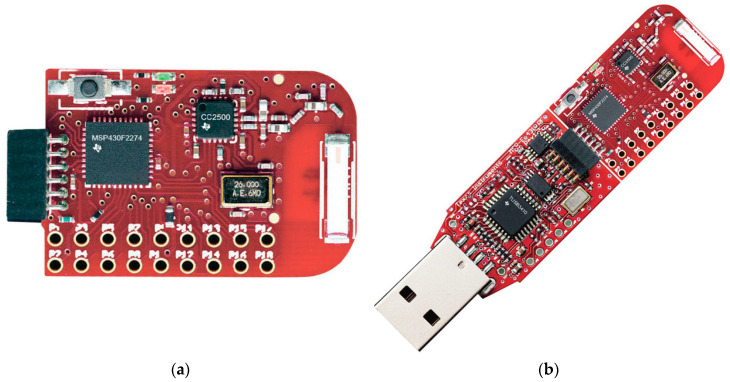
eZ430-RF2500 board components: (**a**) end device, with MSP430 microcontroller, CC2500 wireless transceiver and integrated dielectric antenna; (**b**) access point, identical to the end device, but with attached development board for microcontroller programming and data exchange with PC via USB.

In order to make the instrument truly wearable, with no hampering of the subject mobility, a wireless microcontroller, the Texas Instruments eZ430-RF2500 board(Texas Instruments, Freising, Germany), comprising the MSP430 microcontroller and CC2500 wireless transceiver, is used. The CC2500 chip, in particular, is a high-sensitivity transceiver with low current consumption, designed to operate in the free 2.45 GHz ISM (industrial, scientific and medical) band. It uses a proprietary transmission protocol allowing a maximum data rate of 500 kBaud. It is specifically tailored for low-power applications, showing 400 nA current consumption in sleep mode and providing fast startup times, with a mere 240 µs required to change from sleep to Tx/Rx state. The eZ430-RF2500 board has the additional advantage of providing a ready-to-use platform with microcontroller, transceiver and antenna already integrated on a single board. The eZ430-RF2500 board is constituted by two parts: one, the end device, is connected to the wearable instrument and is fed by the same batteries which supply the PCB performing vital sign detection; the other one, the access point, is connected to a PC and wirelessly collects measurement data transmitted by the end device for subsequent processing, visualization and storage. The two parts are depicted in Figure 4.

The end device, in particular, samples the output signal coming from the acquisition PCB at a rate of 1 ksample/s, using the 10-bit successive approximation analog-to-digital converter (ADC) available onboard the MSP430 microcontroller. It is worth noting that the 10-ms time constant of the envelope detector limits the bandwidth of the output signal to a few tens of hertz, thus making the 1 ksample/s sampling frequency perfectly adequate. The 10-bit samples are encoded using two 8-bit words, giving rise to a data rate of 16 kbit per second, perfectly within the CC2500 capabilities. It is worth noting that, thanks to the novel measurement architecture, as compared to [41], the signal related to cardiac activity has a much higher amplitude, making 10-bit resolution sufficient for signal detection (in the original scheme proposed in [41] a 16-bit resolution was required for detection of the low-level impedance changes due to heart beats). The acquired samples are then transmitted using the CC2500 transceiver 2.45-GHz link, using the SimpliciTI network protocol, and then collected by the access point. The access point is equipped with a programming interface which allows data exchange with the host PC via a USB interface and also provides feeding voltage. However, such interface has a limited baud rate and therefore has been bypassed using directly the internal universal asynchronous receiver-transmitter (UART) of the MSP430 which allows for much higher data rates.

In order to allow ease of use and, at the same time, warrant maximum comfort to the wearer, textile electrodes have been preferred over the pre-gelled electrodes used in the previous system [41]. Among the many conductive textiles available on the market, a Shieldex fabric from Statex has been chosen. The selected conductive textile is composed of 78% polyamide and 22% elastomer, and it is plated by 99%-pure silver. In particular, the silver plating makes the fabric suitable for use in direct contact with the patient skin and the elastomer makes the electrode stretchable. 

The above described hardware has been integrated in a wearable belt, as shown in Figure 3b. The belt is built around a central pocket (which is placed approximately in front of the sternum) which contains the battery holder, the signal acquisition PCB and the Texas Instruments board integrating the microcontroller and the wireless transceiver. Attached to this pocket are four elastic bands with the four textile electrodes sewed with conductive yarn to a snap button which is used to connect the four electrodes to the acquisition PCB. The electrodes of each couple are sewed one on top of the other and their position is such that, once worn, they are placed approximately on the mid-axillary line. The use of snap connections allows for easy removal of the electronic parts for belt washing. The belt is completed by two velcro straps which allow the tight closure of the belt around the subject thorax. The length of the straps has been chosen to allow fitting of thoracic circumferences ranging from 80 to 120 cm.

### 2.4. Signal Processing and User Interface

The signal sampled by the microcontroller and subsequently sent to the access point device contains, as shown in the example in Figure 2b, a superposition of breath and cardiac signals. The signal must be processed to isolate the two components. This process is accomplished through a LabVIEW-based virtual instrument, running on the PC, which retrieves the signal samples from the access point device via USB exploiting the MSP430 UART interface(Texas Intruments, Germany). The signal is then digitally filtered by two digital 5th order Bessel filters: the first one is a low-pass filter with a cut-off frequency of 0.5 Hz, and it isolates the signal component related to breath activity; the second one is a high-pass filter with a cut-off frequency of 1 Hz, and it isolates the ECG signal. It is worth noting that the 1-Hz cutoff has been chosen because the lowest heart rate expected in a healthy adult is 60 bpm, corresponding to 1 Hz fundamental frequency. Even though 1 Hz is higher than the low-cutoff frequency commonly employed in ECG signals, it must be considered that the proposed instrument does not aim at producing a clinical ECG trace, but rather a clean signal allowing a straightforward extraction of the heart rate. The chosen frequency ensures a complete rejection of the breath–activity component and does not compromise the correct heart rate estimation, which is mainly based on QRS detection, where the QRS complex is the ECG component presenting the highest frequency content. The two separated signals are then visualized on two sliding windows, also showing indication of the average breath and heart rates. As a side note, it can be noticed that the signal processing might have been implemented in the wearable part of the instrument. However, this would have increased the computational burden on the MSP430 and would have resulted in early separation of the two signals with consequent need to transfer them separately to the wireless interface. Both these aspects would have resulted in increased power consumption and a consequent decrease in autonomy.

### 2.5. Demonstration of the New Detection Principle and Its Performance

As previously highlighted, one of the improvements of the new belt over the previously developed solution [41], is the ability to directly detect the ECG signal.

In order to show the difference between ECG signal and trans-thoracic impedance changes related to cardiac activity, in terms of signal level and quality, a specific experiment was performed in which a test point was added to the PCB to allow acquisition of the instrumentation amplifier output. The output voltage was acquired with a PXI-6251 DAQ card (National Instruments) mounted on a battery-powered DC PXI chassis. In order to correctly sample the 50-kHz voltage, a sampling rate of 500 ksample/s has been chosen. Please note that the only purpose of this experiment was demonstrating the increase in system performances that can be obtained with the newly developed scheme. However, it does not represent a viable practical solution, because it would require a high-speed digitizer, which would substantially increase the system cost. Therefore, circuit blocks after the instrumentation amplifier are definitely required in the final wearable solution in order to guarantee a low cost solution, allowing the use of much lower sampling frequencies for the envelope-detected signal.

**Figure 5:**
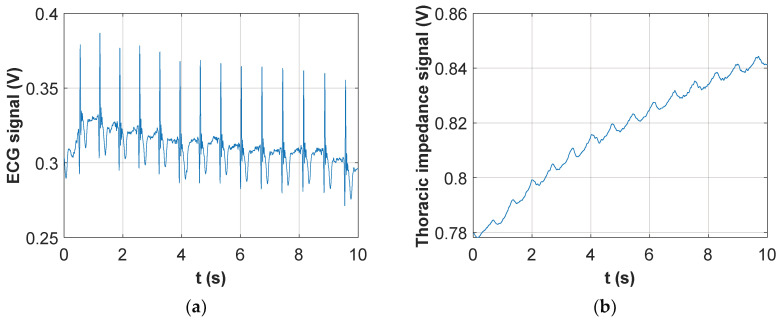
Processed signals from the instrumentation amplifier output: (**a**) Low-pass filtered signal; (**b**) Envelope detection on the high-pass filtered and rectified signal.

The signal acquired in the specific experiment with the DAQ card followed two parallel signal processing paths. In the first processing chain the signal was low-pass filtered with a cut-off frequency of 30 Hz. This removed completely the 50-kHz voltage induced by the injected current and left only low-frequency components. As shown in Figure 5a the resulting signal strictly resembled an ECG. In fact, a parallel acquisition from a custom ECG recording device [41] demonstrated a 1:1 correlation between QRS peaks detected by the two systems.

The second processing chain, instead, performed a high-pass filtering of the signal with a cut-off frequency of 10 kHz. This removed completely the ECG low-frequency components and left the 50-kHz voltage which was modulated by the trans-thoracic impedance variations. The filtered signal was then rectified and its envelope detected, obtaining the output voltage shown in Figure 5b. It is important to highlight that during the 10-s acquisition reported in Figure 4 the subject was holding his breath; therefore, apart from a small low-frequency drift, the periodic changes in the signal were those related to cardiac activity. In fact, it was immediately verified that a dip in the signal was present shortly after each QRS complex detectable in Figure 5a. The trace in Figure 5b was equivalent to those recorded with the instrument previously developed by the authors [41], which relied entirely on impedance measurements. A comparison between Figure 5a,b clearly evidences the great improvement in terms of signal dynamics (from a few mV to several tens of mV) and beat-to-beat time interval detection that was achieved through the direct acquisition of the ECG baseband signal for heart activity monitoring.

Furthermore, these preliminary tests on 10 volunteers carried out employing the DAQ card to sample the instrumentation amplifier output demonstrated that special care must be exerted whilst placing the thoracic belt. If the belt is not tight enough or the electrodes are not placed approximately on the mid-axillary line, it might happen that the cardiac activity is not detectable at all on the basis of the impedance trace. On the other hand, the ECG signal and the respiratory trace were always easily detectable from the acquired signal, with no particular precaution being taken in the belt positioning.

To better demonstrate the above finding, a set of acquisitions on the volunteers was performed, letting the volunteer wear the belt on his own, without special instructions. The result was that, whilst in all cases it was possible to detect both the baseband ECG signal and the impedance variations related to respiratory acts, in 4 out of 10 subjects the cardiac activity was not detectable from the impedance trace, as was immediately apparent from the signal acquired during an apnea phase, which showed a flat impedance trace. Not surprisingly, in these four subjects the EMD technique, successfully applied in [41] to extract heartbeats from the trans-thoracic impedance trace, did not provide any useful result. In the remaining six subjects, however, the first intrinsic mode function (IMF) extracted through EMD conveyed useful information for the extraction of heartbeats and cardiac frequency.

Table 1 shows the comparison, for the six successful subjects, between the average heart rate extracted from the baseband ECG signal and the one retrieved from the first IMF extracted from the impedance trace.

Data in Table 1 show that, when the cardiac activity is correctly detected on the impedance trace, the EMD technique provides excellent performances in terms of heart rate detection, with an average relative error equal to 0.23% and a maximum error of 0.63%.

## 3. Results

After performing the preliminary experiments with the DAQ card acquiring the instrumentation amplifier output, which allowed the demonstration of the detection principle illustrated in Section 2.2, several acquisitions were performed with the developed wearable belt, showing its capabilities in terms of simultaneous cardio–respiratory monitoring.

All results presented in this section have been obtained applying the wearable belt to a set of 10 healthy volunteers (five males and five females), aged between 22 and 45 years, with heights ranging from 155 to 180 cm and weighing between 50 and 80 kg. Informed consent was obtained from each volunteer and experiments were conducted under the direction of a licensed physician.

All participants were requested to sit down for at least 15 minutes before each acquisition, to make sure that traces were acquired in a resting condition. During the subsequent signal acquisition phase, the volunteers were left free to remain sitting or to change to a standing condition, according to their preferred posture. As a result, roughly half of the acquisitions were performed in the standing position and the remaining half in a sitting position. No significant difference was observed in the signal quality between the two different postures. However, it is important to stress that all participants were requested to limit body movements as much as possible, particularly those concerning the upper trunk and the arms. The signal received by the PC with connected access point has been processed by the LabVIEW virtual instrument, which is able to separate the respiratory and heartbeat components.

**Figure 6:**
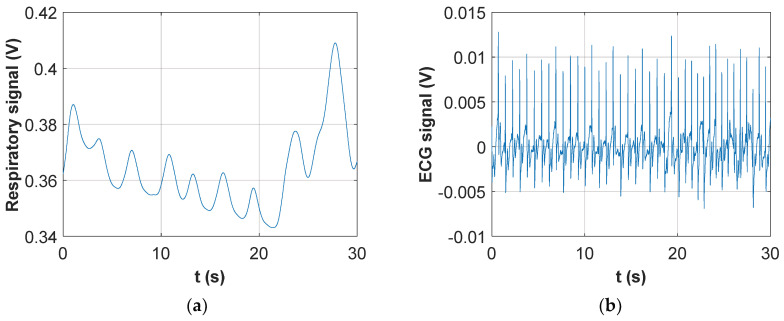
Signals obtained with the wireless wearable belt, after processing through the virtual instrument on the host PC: (**a**) Respiratory signal; (**b**) ECG signal.

As an example, Figure 6 shows, for one of the volunteers, a portion of the two extracted signals.

As evident from the respiratory trace, the subject performed 20 s of normal breathing followed by two deep breaths with increasing inspired volume; the ECG trace in Figure 6b shows how the cardiac signal is perfectly separated from the respiratory one, with no loss of quality in correspondence to the two deep breaths.

### 3.1. Respiratory Monitoring Validation

In order to validate the respiratory trace, a specific recording session was performed for each volunteer, with the subject wearing the belt and simultaneously breathing through the mouthpiece of a portable Spirobank spirometer (Medical International Research—MIR), used as the gold standard. Then, the average breath frequency has been extracted from the respiratory signal acquired through the wearable belt and compared with the one recorded by the spirometer. The average frequency has been extracted from the average breath-to-breath interval, estimated on the basis of the peak-to-peak distance. The portable spirometer only allowed acquisition for a short period of time, and after a validation period of variable length. Therefore, the number of recorded respiratory acts varied from one subject to the other from a minimum of 5 to a maximum of 21 breathing cycles.

The comparison between wearable belt and spirometer is reported in Table 2.

Data in Table 2 show the very good performance in terms of breath rate detection, with an average relative error equal to 1.7% and a maximum error of 4%.

### 3.2. Cardiac Monitoring Validation

In order to validate the cardiac trace, a specific recording session was performed for each volunteer, with the subject wearing the belt and simultaneously attached to a custom ECG recording device, used as the gold standard. As expected, apart from a slight attenuation of the QRS complex due to the limited bandwidth of the trace acquired with the wearable belt, there was an excellent agreement between the RR intervals estimated through the wearable belt and the ECG device. The differences were totally negligible (within a few milliseconds), mostly arising from the employed sampling frequency of 1 kHz, providing a time resolution of 1 ms.

These recording sessions were also used to assess the performance of the EMD technique, as an alternative to filtering, for separating the ECG signal from the respiratory one. Indeed, in the previous version of the instrument [41], EMD was proven a suitable technique for the purpose. However, in the previous work the technique was applied on the impedance trace, where changes related to cardiac activity were the ones with the smallest time variation scale and, therefore, they were immediately obtained from the first intrinsic mode function (IMF), representing the highest-frequency component of the signal. In the new instrument version, instead, the whole ECG signal is present, which has a much more complex shape with different time scales. Therefore, it was found that the most suitable IMF for extracting the heartbeat information must be manually selected by visual inspection. Moreover, the extracted IMF has a fairly variable peak height, strongly changing from subject to subject, making the choice of a universal threshold for finding signal peaks more difficult.

**Figure 7:**
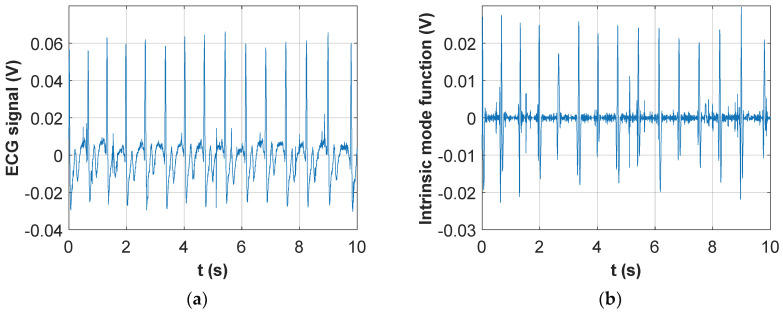
Cardiac signal obtained with the wireless wearable belt, after processing through the virtual instrument on the host PC: (**a**) Low-pass filtering; (**b**) Intrinsic mode function selection.

To illustrate the above findings, Figure 7 shows the comparison, for 10 s of recording extracted from one of the ten subjects, between the ECG signal obtained through filtering and the most appropriate IMF (which, in this case, turned out to be the second one).

Comparison between Figure 7a,b shows that the correct IMF, which must be manually selected among those computed by the EMD technique, detects a peak for each QRS complex. However, there are also some spurious peaks that must be filtered out by choosing an appropriate threshold. Altogether, the whole technique becomes much too complex to be used in an automated environment. Nonetheless, once due attention was paid to the selection of the correct IMF and of an adequate threshold for peak finding, the average heart rates extracted from the ECG signal retrieved through simple filtering, or from the identified IMF, showed excellent agreement with average and peak errors well below 1%.

## 4. Discussion

The results obtained through the tests carried out sampling the instrumentation amplifier output with a high-end DAQ card demonstrated the working hypothesis that the envelope detection method, which could be easily implemented in a portable and low-cost version, was able not only to detect trans-thoracic impedance changes, but also to directly retrieve the ECG signal. This dual-sensing capability considerably improved performances in heart rate detection, as compared to a purely impedance-based scheme. Indeed, the heart rate detection proved to be much more reliable, without requiring the observance of particular precautions in thoracic belt wearing, and the heartbeat extraction from the acquired signal could be accomplished in a straightforward manner by simple filtering. This helps to make the signal processing requirements less demanding, as compared to the EMD solution adopted for the impedance trace. In terms of measurement accuracy, the heart rate detection with the two techniques, i.e. direct ECG detection and extraction from trans-thoracic impedance trace, showed similar performances but with the need to take special care in the belt positioning for the second technique.

The results obtained with the wearable and wireless instrument highlighted the excellent performance of the developed solution, with respiratory rate estimation showing average errors around 2% as compared to a portable spirometer, used as a gold standard. Thanks to the direct acquisition of the ECG signal, the performance in heart rate detection is even better, with results in perfect agreement with those achieved through a two-lead ECG recording device. This is a major improvement over the previous version [41], entirely based on trans-thoracic impedance, where an average error around 3% was present in heart rate estimates.

Results shown in [32], where a smart textile FBG sensor is used, present a maximum error of 0.38% for the respiration, but this was monitored only in standing and supine positions. This result demonstrates an improvement in the relative error as compared to previous prototypes [33,34]. In [35] the fiber optic-based system was assessed only in supine position and it is not wearable, while in [36] measurement error analysis was not performed. A metrological analysis, instead, is reported in [4], where a novel piezoelectric-based approach is investigated and errors lower than 5% were found counting the heartbeat and the breaths taken in one minute. However, comparing the sensor with conventional equipment and methods the average error ± STD (%) is 10 ± 6 and 6 ± 3.5 in respiration and heartbeat rate, respectively. Performances of the system shown in [38] by conducting overnight experiments are very promising, indicating that simultaneous measurements of ECG signals, including T wave and QT interval measurements, and respiratory signals were possible. However, this is true only if individuals maintained ordinary body positions such as supine position and left and right lateral positions. 

Also based on the literature comparison, the developed instrument shows excellent performances for respiratory and heart rates estimation, is totally wearable and unobtrusive, and can be realized with off-the-shelf components at a price largely below EUR 100. The current consumption, excluding the CC2500 wireless interface, is around 2.5 mA, which would provide enough autonomy to allow for 24-h monitoring of people. The actual duration with the AAA alkaline batteries, however, is currently limited to around 8 hours, due to the high consumption from the wireless interface; indeed, due to the relatively high bit rate as compared to common sensor node applications, the CC2500 works with a very high duty cycle (the low-power sleep state is hardly entered). If there is no need to perform real-time monitoring of the cardio–respiratory activity, the wireless interface might be removed altogether and the acquired signal samples stored on a SD memory for subsequent retrieval. This would enhance battery duration to several days.

## 5. Conclusions

A fully wearable thoracic belt with embedded textile electrodes has been developed, suitable for simultaneously monitoring respiratory and cardiac frequencies.

The system is based on a four-electrode scheme, with a couple of electrodes injecting a 50-kHz sinusoidal current in the patient thorax and an instrumentation amplifier acquiring the trans-thoracic voltage at the second couple of electrodes. By applying a simple envelope detection technique at the sensed voltage, a signal containing both the ECG component and the breath-modulated impedance is obtained. By suitable low-pass and high-pass filtering of this signal, the respiratory and heart activities, and related frequencies, are straightforwardly extracted. In spite of the extreme simplicity and low cost of the system, the obtained performances in terms of cardio–respiratory rate estimation are comparable to gold-standard spirometry and 2-lead ECG techniques.

Even though the developed wireless interface makes the system potentially suitable for monitoring during everyday or even sports activities, at the moment no specific provision is made for the removal of motion artefacts and, hence, the system can be used only for subjects in an almost stationary position.

However, various algorithms and techniques are available in the literature for motion artifact removal [48] and their integration in the proposed system will be the subject of future work.

Moreover, improvements are also foreseen in the belt configuration, with the possible addition of a layer of foam rubber beneath the textile electrodes to ensure a better adherence to the skin, even when the belt is not tightly fastened.

## Ethical Statement

All subjects gave their informed consent for inclusion before they participated in the study.

## Figures and Tables

**Table 1:** Comparison between average heart rates obtained from the baseband ECG signal and from the trans-thoracic impedance trace via empirical mode decomposition (EMD).

Subject #	Average Heart Rate (ECG)	Average Heart Rate (IMF)	Relative Error (Absolute Value)
1	61.17 bpm	61.15 bpm	0.03%
2	62.22 bpm	62.18 bpm	0.06%
3	76.80 bpm	76.97 bpm	0.22%
4	93.56 bpm	93.23 bpm	0.35%
5	97.61 bpm	97.54 bpm	0.07%
6	94.30 bpm	94.90 bpm	0.63%

**Table 2:** Comparison between average respiratory rates obtained with the wearable belt and the portable spirometer.

Subject #	Average Breath Rate (Belt)	Average Breath Rate (Spirometer)	Relative Error (Absolute Value)
1	18.34 bpm	18.73 bpm	2.1%
2	10.29 bpm	10.72 bpm	4.0%
3	12.62 bpm	12.68 bpm	0.5%
4	15.67 bpm	15.97 bpm	1.9%
5	22.49 bpm	22.12 bpm	1.7%
6	21.30 bpm	21.77 bpm	2.2%
7	32.16 bpm	32.65 bpm	1.5%
8	24.13 bpm	24.31 bpm	0.7%
9	20.12 bpm	20.32 bpm	1.0%
10	28.85 bpm	28.36 bpm	1.7%

## References

[1] Pisa S., Pittella E., Piuzzi E. (2016). A survey of radar systems for medical applications. IEEE Aerosp. Electron. Syst. Mag..

[2] Baig M.M., Gholamhosseini H., Connolly M.J. (2013). A comprehensive survey of wearable and wireless ECG monitoring systems for older adults. Med. Biol. Eng. Comput..

[3] Lo Presti D., Romano C., Massaroni C., D’Abbraccio J., Massari L., Caponero M.A., Oddo C.M., Formica D., Schena E. (2019). Cardio-respiratory monitoring in archery using a smart textile based on flexible fiber Bragg grating sensors. Sensors.

[4] Allataifeh A., Al Ahmad M. (2020). Simultaneous piezoelectric noninvasive detection of multiple vital signs. Sci. Rep..

[5] Van Loon K., Van Zaane B., Bosch E.J., Kalkman C.J., Peelen L.M. (2015). Non-invasive continuous respiratory monitoring on general hospital wards: A systematic review. PLoS ONE.

[6] Mishra A., McDonnell W., Wang J., Rodriguez D., Li C. (2019). Intermodulation-based nonlinear smart health sensing of human vital signs and location. IEEE Access.

[7] Zhang T., Sarrazin J., Valerio G., Istrate D. (2018). Estimation of human body vital signs based on 60 GHz Doppler radar using a bound-constrained optimization algorithm. Sensors.

[8] Bernardi P., Cicchetti R., Pisa S., Pittella E., Piuzzi E., Testa O. (2013). Design, realization, and test of a UWB radar sensor for breath activity monitoring. IEEE Sens. J..

[9] Lai J.C.Y., Xu Y., Gunawan E., Chua E.C.P., Maskooki A., Guan Y.L., Low K.S., Soh C.B., Poh C.L. (2010). Wireless sensing of human respiratory parameters by low-power ultrawideband impulse radio radar. IEEE Trans. Instrum. Meas..

[10] Sacco G., Piuzzi E., Pittella E., Pisa S. (2020). An FMCW radar for localization and vital signs measurement for different chest orientations. Sensors.

[11] Wang G., Gu C., Inoue T., Li C. (2014). A hybrid FMCW-interferometry radar for indoor precise positioning and versatile life activity monitoring. IEEE Trans. Microw. Theory Techn..

[12] Pittella E., Zanaj B., Pisa S., Cavagnaro M. (2017). Measurement of breath frequency by body-worn uwb radars: A comparison among different signal processing techniques. IEEE Sens. J..

[13] Pantelopoulos A., Bourbakis N.G. (2010). A survey on wearable sensor-based systems for health monitoring and prognosis. IEEE Trans. Syst. Man Cybern. Part C Appl. Rev..

[14] Manujło A., Osuch T. Temperature fiber Bragg grating based sensor for respiration monitoring. Proceedings of the Photonics Applications in Astronomy, Communications, Industry, and High Energy Physics Experiments 2017.

[15] Issatayeva A., Beisenova A., Tosi D., Molardi C. (2020). Fiber-optic based smart textiles for real-time monitoring of breathing rate. Sensors.

[16] Krehel M., Schmid M., Rossi R.M., Boesel L.F., Bona G.L., Scherer L.J. (2014). An optical fibre-based sensor for respiratory monitoring. Sensors.

[17] Li X., Liu D., Kumar R., Ng W.P., Fu Y.Q., Yuan J.H., Yu C.X., Wu Y.F., Zhou G.R., Farrell G. (2017). A simple optical fiber interferometer based breathing sensor. Meas. Sci. Technol..

[18] Massaroni C., Di Tocco J., Lo Presti D., Longo U.G., Miccinilli S., Sterzi S., Formica D., Saccomandi P., Schena E. (2019). Smart textile based on piezoresistive sensing elements for respiratory monitoring. IEEE Sens. J..

[19] Balakrishnan V., Dinh T., Foisal A.R.M., Nguyen T., Phan H.P., Dao D.V., Nguyen N.T. (2019). Paper-based electronics using graphite and silver nanoparticles for respiration monitoring. IEEE Sens. J..

[20] Min S.D., Yun Y., Shin H. (2014). Simplified structural textile respiration sensor based on capacitive pressure sensing method. IEEE Sens. J..

[21] Mellal I., Laghrouche M., Bui H.T. (2017). Field programmable gate array (FPGA) respiratory monitoring system using a flow microsensor and an accelerometer. Meas. Sci. Rev..

[22] Janidarmian M., Fekr A.R., Radecka K., Zilic Z. (2017). Multi-objective hierarchical classification using wearable sensors in a health application. IEEE Sens. J..

[23] Koch E., Dietzel A. (2016). Skin attachable flexible sensor array for respiratory monitoring. Sens. Actuators A Phys..

[24] Yang J., Chen B., Zhou J., Lv Z. (2015). A low-power and portable biomedical device for respiratory monitoring with a stable power source. Sensors.

[25] Janik P., Janik M.A., Wróbel Z. (2016). Integrated micro power frequency breath detector. Sens. Actuators A Phys..

[26] André N., Druart S., Dupuis P., Rue B.D., Gérard P., Flandre D., Raskin J., Francis L.A. (2012). Dew-based wireless mini module for respiratory rate monitoring. IEEE Sensors J..

[27] Jiang P., Zhao S., Zhu R. (2015). Smart sensing strip using monolithically integrated flexible flow sensor for noninvasively monitoring respiratory flow. Sensors.

[28] Padasdao B., Shahhaidar E., Stickley C., Boric-Lubecke O. (2013). Electromagnetic biosensing of respiratory rate. IEEE Sens. J..

[29] Rajanna R.R., Sriraam N., Vittal P.R., Arun U. (2020). Performance evaluation of woven conductive dry textile electrodes for continuous ECG signals acquisition. IEEE Sens. J..

[30] Fontana P., Martins N.R.A., Camenzind M., Boesch M., Baty F., Schoch O.D., Brutsche M.H., René M., Rossi R.M., Annaheim S. (2019). Applicability of a textile ECG-belt for unattended sleep apnoea monitoring in a home setting. Sensors.

[31] Tang Y., Chang R., Zhang L., Yan F., Ma H., Bu X. (2020). Electrode humidification design for artifact reduction in capacitive ECG measurements. Sensors.

[32] Lo Presti L., Massaroni C., Formica D., Saccomandi P., Giurazza F., Caponero M.A., Schena E. (2017). Smart textile based on 12 fiber Bragg gratings array for vital signs monitoring. IEEE Sens. J..

[33] Massaroni C., Saccomandi P., Formica D., Lo Presti D., Caponero M.A., Di Tomaso G., Francesco Giurazza F., Muto M., Schena E. (2016). Design and feasibility assessment of a magnetic resonance-compatible smart textile based on fiber Bragg grating sensors for respiratory monitoring. IEEE Sens. J..

[34] Chen Z., Lau D., Teo J.T., Ng S.H., Yang X., Kei P.L. (2014). Simultaneous measurement of breathing rate and heart rate using a microbend multimode fiber optic sensor. J. Biomed. Opt..

[35] Krej M., Dziuda Ł., Skibniewski F.W. (2015). A method of detecting heartbeat locations in the ballistocardiographic signal from the fiberoptic vital signs sensor. IEEE J. Biomed. Health Inform..

[36] Silva A.F., Carmo J.P., Mendes P.M., Correia J.H. (2011). Simultaneous cardiac and respiratory frequency measurement based on a single fiber Bragg grating sensor. Meas. Sci. Technol..

[37] Takano M., Yamagishi S., Ohmuta T., Fukuoka Y., Ueno A. (2017). Non-contact simultaneous measurements of electrocardiogram and respiratory movements using capacitive sheet electrodes. Adv. Biomed. Eng..

[38] Trindade I.G., Machado da Silva J., Miguel R., Pereira M., Lucas J., Oliveira L., Valentim B., Barreto J., Santos Silva M. (2016). Design and evaluation of novel textile wearable systems for the surveillance of vital signals. Sensors.

[39] Khalil S.F., Mohktar M.S., Ibrahim F. (2014). The theory and fundamentals of bioimpedance analysis in clinical status monitoring and diagnosis of diseases. Sensors.

[40] Pittella E., Piuzzi E., Rizzuto E., Pisa S., Del Prete Z. (2018). Metrological characterization of a combined bio-impedance plethysmograph and spectrometer. Measurement.

[41] Piuzzi E., Pisa S., Pittella E., Podestà L., Sangiovanni S. (2019). Low-cost and portable impedance plethysmography system for the simultaneous detection of respiratory and heart activities. IEEE Sens. J..

[42] Thiele R.H., Bartels K., Gan T.J. (2015). Cardiac output monitoring: A contemporary assessment and review. Crit. Care Med..

[43] Irzma´nska E., Padula G., Irzma´nski R. (2014). Impedance plethysmography as a tool for assessing exertion-related blood flow changes in the lower limbs in healthy subjects. Measurement.

[44] Trobec R., Rashkovska A., Avbelj V. (2012). Two proximal skin electrodes—A respiration rate body sensor. Sensors.

[45] Labate D., Foresta F.L., Occhiuto G., Morabito F.C., Lay-Ekuakille A., Vergallo P. (2013). Empirical mode decomposition vs. wavelet decomposition for the extraction of respiratory signal from single-channel ECG: A comparison. IEEE Sens. J..

[46] Pinheiro E., Postolache O., Girão P. (2012). Empirical mode decomposition and principal component analysis implementation in processing non-invasive cardiovascular signals. Measurement.

[47] (2005). Medical Electrical Equipment—Part. 1: General Requirements for Basic Safety and Essential Performance.

[48] Postolache O., Girão P., Mendes J., Pinheiro E.C., Postolache G. (2010). Physiological parameters measurement based on wheelchair embedded sensors and advanced signal processing. IEEE Trans. Instrum. Meas..

